# Emergence of SARS-CoV-2 Delta Variant and Effect of Nonpharmaceutical Interventions, British Columbia, Canada

**DOI:** 10.3201/eid2910.230055

**Published:** 2023-10

**Authors:** Y.L. Elaine Chan, Michael A. Irvine, Natalie Prystajecky, Hind Sbihi, Marsha Taylor, Yayuk Joffres, Andrea Schertzer, Caren Rose, Louise Dyson, Edward M. Hill, Michael Tildesley, John R. Tyson, Linda M.N. Hoang, Eleni Galanis

**Affiliations:** British Columbia Centre for Disease Control, Vancouver, British Columbia, Canada (Y.L.E. Chan, M.A. Irvine, N. Prystajecky, H. Sbihi, M. Taylor, Y. Joffres, A. Schertzer, C. Rose, J.R. Tyson, L.M.N. Hoang, E. Galanis);; Public Health Agency of Canada, Ottawa, Ontario, Canada (Y.L.E. Chan, A. Schertzer);; Simon Fraser University, Burnaby, British Columbia, Canada (M.A. Irvine);; University of British Columbia, Vancouver (N. Prystajecky, H. Sbihi, C. Rose, L.M.N. Hoang, E. Galanis);; University of Warwick, Coventry, UK (L. Dyson, E.M. Hill, M. Tildesley);; Joint UNIversities Pandemic and Epidemiological Research (L. Dyson, E.M. Hill, M. Tildesley)

**Keywords:** COVID-19, SARS-CoV-2, B.1.617.2 SARS-CoV-2 variant, SARS-CoV-2 Delta variant, communicable disease control, disease transmission, infectious, vaccination, physical distancing, public health, severe acute respiratory syndrome coronavirus 2, viruses, respiratory infections, zoonoses, British Columbia, Canada

## Abstract

In British Columbia, Canada, initial growth of the SARS-CoV-2 Delta variant was slower than that reported in other jurisdictions. Delta became the dominant variant (>50% prevalence) within ≈7–13 weeks of first detection in regions within the United Kingdom and United States. In British Columbia, it remained at <10% of weekly incident COVID-19 cases for 13 weeks after first detection on March 21, 2021, eventually reaching dominance after 17 weeks. We describe the growth of Delta variant cases in British Columbia during March 1–June 30, 2021, and apply retrospective counterfactual modeling to examine factors for the initially low COVID-19 case rate after Delta introduction, such as vaccination coverage and nonpharmaceutical interventions. Growth of COVID-19 cases in the first 3 months after Delta emergence was likely limited in British Columbia because additional nonpharmaceutical interventions were implemented to reduce levels of contact at the end of March 2021, soon after variant emergence.

Throughout the COVID-19 pandemic, SARS-CoV-2 variants have emerged through viral mutation. Variants demonstrating an increase in transmissibility or virulence; changes in clinical manifestations; or a decrease in the effectiveness of public health measures, diagnostics, vaccines, or therapeutics are designated variants of concern (VOCs) by the World Health Organization (*1*). By June 2021, a total of 4 SARS-CoV-2 variants had been designated VOCs (*1*). Designated a VOC in May 2021, Delta largely replaced the earlier Alpha, Beta, and Gamma VOCs because of its comparatively higher transmissibility (*2*). In India, where it was first detected, Delta outcompeted Alpha and drove an increase of COVID-19 cases beginning in March 2021 (*3*). By mid-August 2021, Delta represented >90% of genetically sequenced SARS-CoV-2 samples submitted to GISAID (https://www.gisaid.org), dominating on a global scale until its decline in favor of Omicron beginning in December 2021 (*4*,*5*).

By March 1, 2021, British Columbia (2021 population 5,214,805), Canada, had reported >80,000 COVID-19 cases and detected Alpha, Beta, and Gamma VOC cases among residents (*6*). The Delta VOC was first detected in British Columbia during the week of March 21, 2021, but did not grow to dominance (>50% prevalence) until 17 weeks later, during the week of July 18, 2021, after major relaxations in public health measures, or nonpharmaceutical interventions (NPIs).

Differences in factors such as NPIs, vaccination rates, competing variants in circulation, and population density and behavior may result in interjurisdictional differences in the transmission and growth rates of variants (*7*–*10*). The initial growth and time to dominance of Delta in British Columbia was slower than in jurisdictions such as England, Scotland, and several US states (*7*–*10*). In England, Delta grew to dominance ≈10 weeks after the fifth case was detected in mid-March 2021, reaching 62% prevalence among sequenced cases by mid-May 2021 (*7*). In Scotland, the Delta VOC rapidly replaced Alpha during April–May 2021 (*8*,*9*), and across 6 states in the United States, the average time from first detection of Delta to its dominance was ≈10 weeks (71 days, range 54–92 days) (*10*).

British Columbia and England are adequately comparable because they have universal healthcare systems, similar population age distribution (median age 42.8 years in British Columbia, 40 years for England and Wales in 2021), and similar temperate climates within the main metropolitan areas. Of note, key differences existed in public health policy and vaccination coverage between British Columbia and England around the time of Delta emergence; England ultimately experienced both a shorter time to dominance for Delta and higher subsequent growth in COVID-19 incidence (Appendix Figure 1). In British Columbia, circuit-breaker NPIs, including restricting travel outside the region of residence unless essential; suspending indoor dining, worship services, and adult group fitness activities; and expanding mask requirements in schools to younger age groups, were implemented on March 30, 2021, shortly after Delta variant was detected, in response to rising numbers of Alpha and Gamma variant cases (*11*). Those measures supplemented existing NPIs, which required physical distancing and masks in all public indoor settings, restricted gatherings, and encouraged workplaces to adopt remote working conditions (Appendix Table 1). During March–June 2021, the 7-day rolling COVID-19 incidence rate per 100,000 population in British Columbia peaked at 21.8 in mid-April 2021 before decreasing to a low of 1.0 at the end of June 2021.

Conversely, in the time surrounding Delta introduction and initial growth, England was in the early stages of reopening after lockdown and had begun gradually relaxing measures, including reopening schools to all students, replacing a stay-at-home order with a recommendation to stay local, and stepwise reopening of businesses and public buildings (*12*) (Appendix Table 1). England observed an initial decrease in its 7-day rolling COVID-19 incidence rate per 100,000 population from 77.0 in March 2021 to 20.7 for the beginning of May 2021, before seeing a substantial increase driven by Delta to 229.1 by the end of June 2021 (*13*) (Appendix Figure 1).

However, population COVID-19 vaccine coverage also differed between British Columbia and England; British Columbia had higher coverage among younger age groups (*14*,*15*). COVID-19 vaccination coverage overall and for persons >45 years of age were lower in British Columbia than in England during March–June 2021, but rates of first-dose coverage for persons 18–34 years of age in British Columbia exceeded those in England by May 2021 (Appendix Figure 2, panel A). Another key difference was the vaccine product used: most vaccine doses administered during March–June 2021 in British Columbia were the mRNA-based BNT162b2 (Pfizer-BioNTech, https://www.pfizer.com) or mRNA-1273 (Moderna, https://www.modernatx.com), and most administered in England were ChAdOx1 (Oxford-AstraZeneca, https://www.astrazeneca.com) (*16*).

The first objective of this study was to describe the emergence of the Delta VOC in British Columbia with respect to the presence of competing variants and case demographics, vaccination status, and travel history. The second objective was, through counterfactual modeling, to identify the main factors for the initially low rate of COVID-19 transmission in British Columbia after Delta variant introduction. Using England as the counterfactual scenario because of its similarities with British Columbia and the availability of public data from UK Health Security Agency, we explored the effects of differences in the proportion of Delta among all infections, public health measures, and vaccine coverage and type on the modeled number of overall COVID-19 cases in British Columbia.

## Methods

### SARS-CoV-2 Lineage Data

In British Columbia, SARS-CoV-2 quantitative PCR (qPCR) testing is offered by hospitals, private laboratories, and the British Columbia Centre for Disease Control (BCCDC) Public Health Laboratory (PHL), which serves as the reference laboratory for the province; VOC monitoring is performed primarily by the BCCDC PHL. During March 1–May 29, 2021 (US Centers for Disease Control and Prevention epidemiologic weeks [epiweeks] 9–21), a combined VOC testing strategy using both screening (i.e., targeted VOC single-nucleotide variant qPCR) and whole-genome sequencing (WGS) was applied to monitor VOC prevalence in BC (Appendix Table 2). During this period, the weekly percentage of samples undergoing VOC screening ranged from 80%–99% and the percentage undergoing WGS ranged from 31%–79%. During May 30–June 30, 2021 (epiweeks 22–26), WGS was attempted for all samples; 69%–79% of all weekly positive samples were successfully sequenced. VOC case definitions are provided (Appendix Table 3).

For samples that underwent both VOC screening and WGS, we used lineage results from WGS. We included only samples with >85% sequence coverage and no quality control flags in ncov-tools (https://github.com/jts/ncov-tools). We classified cases as having unknown lineage if samples did not undergo VOC screening or WGS, were screened VOC-negative or indeterminate and did not undergo WGS, or were not screened and failed WGS.

### Study Population

We linked COVID-19 case investigation and SARS-CoV-2 lineage data by using the patient’s full name, date of birth, and personal health number. We performed linkage using SAS version 9.4 (SAS Institute Inc., https://www.sas.com). We included all COVID-19 cases reported in British Columbia with case investigation information and specimen collection during March 1–June 30, 2021. For records with multiple specimen collection dates, we used the earliest positive date. For cases missing specimen collection date (n = 2,637; 4.0% of final study population), we used symptom onset date, followed by date of case report to the regional health authority. We performed data cleaning, analysis, and figure creation using R version 3.5.2 (The R Foundation for Statistical Computing, https://www.r-project.org).

Travel history information was collected during routine case investigation. Information on international travel was supplemented by reason for testing recorded in the BCCDC PHL database (e.g., international arrivals testing). Delta variant case-patients who had a travel history outside British Columbia were assumed to have acquired infection outside the province; those cases were considered Delta introductions.

COVID-19 vaccination status at time of case detection was linked from British Columbia’s Provincial Immunization Registry using case identifiers. We considered case-patients fully vaccinated if symptom onset (or positive specimen collection if the onset date was not available) occurred >14 days after the second dose of BNT162b2, mRNA-1273, or ChAdOx1; additional doses were not yet approved or recommended during the study period. Case-patients were considered partially vaccinated if they were not fully vaccinated and onset or specimen collection occurred >21 days after first dose. Case-patients without any recorded vaccination or with onset or specimen collection <21 days after the first dose were considered unvaccinated.

### Counterfactual Modeling Methods

We implemented counterfactual modeling using an established model of COVID-19 transmission dynamics in British Columbia (*17*). The model is an adapted susceptible-exposed-infected-recovered compartmental ordinary differential equation model. Additional modeled compartments included a quarantine compartment and a proportion of the population that participate in social distancing with analogous susceptible-exposed-infected-recovered compartments for the social distancing group. We used a Bayesian statistical model in the inference of the basic reproductive number, the fraction change in social distancing between predefined breakpoints, and a dispersion parameter associated with a negative binomial term to observed cases (*17*). We explored differences in the following factors on the modeled number of COVID-19 cases in British Columbia (Appendix Table 4).

#### Proportion of Delta variant Among All Infections

We extracted weekly data on the proportion of the Delta variant among cases in England from the July 23, 2021, UK Health Security Agency report (*18*) and included the proportion of all cases that were genotyped. Unlike in British Columbia, the proportion of cases of Delta in England transitioned from <5% to >80% during May–June 2021 (Appendix Figure 2, panel B). We incorporated logistic functions representing the relative proportion of Delta to represent the relative differences in growth between jurisdictions. We directly incorporated that function into the time-varying transmission term for each scenario, representing the per-contact transmissibility increasing in proportion to the changing composition of variants. Because we used the sampled proportion of Delta variant as input in the modeling, we did not directly explore reasons for their differences between jurisdictions within these scenarios.

#### Levels of Contact Leading to Transmission, Guided by Changes in NPIs

We constructed the transmission scenario for England on the basis of the fitted transmission estimate for British Columbia. We applied an increase in transmission rate to the England scenario after the March 30, 2021, circuit breaker measures were implemented in British Columbia, considering that those NPIs likely led to a reduction in cases in British Columbia but similar measures were not in place in England (Appendix Figure 1).

#### Vaccination Coverage and Majority Vaccine Product Administered

Data on age-dependent vaccination coverage extracted from the UK Government COVID-19 dashboard (*15*) (for England) and the Provincial Immunization Registry (for British Columbia) included vaccination coverage by number of doses (1 or 2) and by age group (12–17 years, 18–24 years, 10-year bands for 25–74 years, and >75 years) (Appendix Figure 2, panel A). We derived parameters for estimated vaccine efficacy on the Delta variant by product and dose on the basis of previous values (Appendix Table 5) (*12*). To account for differences in vaccination scheduling, we collected data on proportion of vaccine coverage by first and second dose by age group from both jurisdictions and weighted them for British Columbia’s population.

We fitted the model using a variational Bayes approach (*17*,*19*) to reported case data for British Columbia during March 1, 2020–July 12, 2021, with 4 transmission segments covering the study period, starting on January 25, March 29, April 5, and May 25, 2021 (Appendix Table 4). This work was conducted under the public health surveillance mandate of the BCCDC, and institutional review board approval was not sought. The planning, conduct, and reporting of this study was in line with the Declaration of Helsinki, as revised in 2013.

## Results

### Delta VOC Emergence

**Figure 1:**
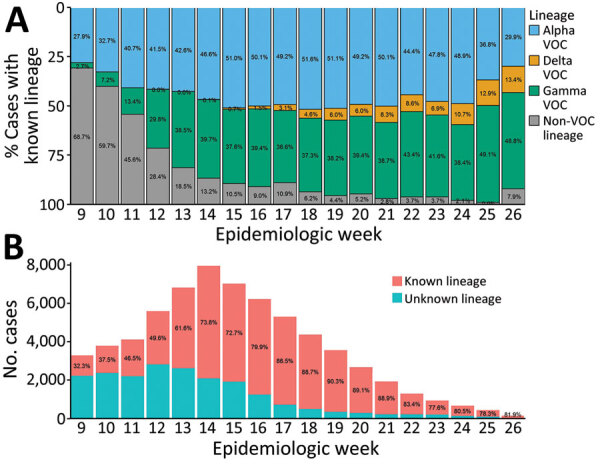
Percentage of COVID-19 cases by SARS-CoV-2 VOC lineage (A) and by known versus unknown lineage (B) reported in British Columbia, Canada, by epidemiologic week of specimen collection, March 1–June 30, 2021 (n = 66,247). Data are incomplete for epiweeks 9 and 26 because of study period date cutoffs. Beta VOC cases are not displayed in the top panel but are accounted for in the rounded percentages; Beta VOC cases did not account for >20 cases (<1% of cases with known lineage) per week in BC during the study period. COVID-19 cases of unknown lineage included cases with samples that did not undergo targeted VOC single-nucleotide variant (SNV) quantitative PCR (qPCR) screening or whole-genome sequencing (WGS), were negative or indeterminate on VOC SNV qPCR screening and did not undergo WGS, or did not undergo VOC SNV qPCR screening and failed WGS. VOC, variant of concern.

A total of 66,247 COVID-19 cases were reported in British Columbia during March 1–June 30, 2021; of those, 1,178 (1.8%) were Delta, 37,872 (57.2%) were other VOCs (Alpha, Beta, or Gamma), 6,930 (10.4%) were non-VOC, and 20,267 (30.6%) were of unknown lineage. During the study period, Alpha and Gamma were the most prevalent variants in British Columbia, codominating from April (epiweek 13) onwards; Alpha reached 46.1% of weekly incident cases (51.6% of cases with known lineage) during May 2–15 (epiweeks 18 and 19), and Gamma reached 40.0% of incident cases (48.8% of cases with known lineage) by the end of June 2021 (epiweek 26) (Figure 1). The prevalence of Beta was negligible, accounting for <20 incident cases (<0.3%) per week.

The Delta VOC was first detected in British Columbia during epiweek 12; the earliest detected case had a specimen collection date of March 21, 2021. Delta case-patients were generally young (65.7% <40 years of age), and a slightly higher percentage were male than female (Table 1). Most Delta variant cases were in unvaccinated persons (83.2%), and most (86.4%) were identified as Pangolin (*20*) lineage B.1.617.2. The prevalence of the Delta variant reached its highest point at the end of the study period; during the last full epiweek (epiweek 25), 44 Delta cases occurred, which represented 10.1% of incident cases (12.9% of cases with known lineage) (Figure 1).

**Table 1:** Pangolin lineage and patient demographics, vaccination status, and travel history for 1,178 SARS-CoV-2 Delta variant of concern cases reported in British Columbia, Canada, March 1–June 30, 2021

Characteristic	No. (%) Delta cases
Pangolin lineage*	
B.1.617.2	1,018 (86.4)
AY.18	44 (3.7)
AY.15	30 (2.5)
AY.10	22 (1.9)
AY.93	20 (1.7)
Other AY lineages	44 (3.7)
Patient demographics	
Age group, y	
<20	261 (22.2)
20–39	513 (43.5)
40–59	248 (21.1)
60–79	118 (10.0)
>80	38 (3.2)
Sex	
F	553 (46.9)
M	622 (52.8)
Unknown	3 (0.3)
Vaccination status†	
Fully vaccinated	30 (2.5)
Partially vaccinated	168 (14.3)
Unvaccinated	980 (83.2)
Travel history	
International travel	153 (13.0)
Domestic travel outside BC	14 (1.2)
No known travel or only within BC	1011 (85.8)

*Pangolin version 4.0.5, Usher version 1.6, Pango version 1.6 (*20*). BC, British Columbia. †Unadjusted proportions; vaccination status shown was at time of symptom onset or specimen collection. Patients were considered fully vaccinated if symptom onset (or positive specimen collection date if onset not available) occurred >14 d after second dose of BNT162b2 (Pfizer, https://www.pfizer.com), mRNA-1273 (Moderna, https://www.modernatx.com), or ChAdOx1 (AstraZeneca, https://www.astrazeneca.com). Patients were considered partially vaccinated if not fully vaccinated and onset/specimen collection date occurred >21 d after first dose. Persons without any recorded vaccine dose or with onset/specimen collection date <21 d after first dose were considered unvaccinated.

**Figure 2:**
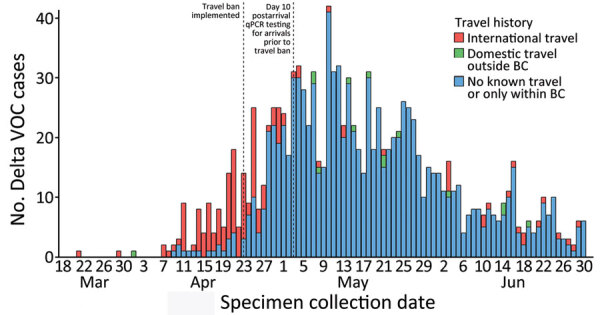
Epidemiologic curve of SARS-CoV-2 Delta VOC cases in British Columbia, Canada, by patient travel history, March 1–June 30, 2021 (n = 1,178). Delta VOC cases classified as having no known travel include 8 cases with missing travel information. Effective at 8:30 p.m. on April 22, 2021 (labeled as April 23 on figure), the government of Canada implemented a ban on all direct commercial and private passenger flights from India and Pakistan (*21*). The travel ban on direct flights from India remained in effect for the rest of the study period, whereas the ban on direct flights from Pakistan was in effect until June 21, 2021 (*21*,*22*). Travelers arriving before the federal travel ban were required to complete day 10 postarrival qPCR testing; as a result, travelers arriving before April 23 might have had specimens collected up to May 2, 2021. BC, British Columbia; qPCR, quantitative PCR; VOC, variant of concern.

Overall, 14.2% (n = 167) of Delta case-patients had known history of travel outside BC; 91.6% (n = 153) had traveled internationally and 8.4% (n = 14) had traveled only within Canada. On the evening of April 22, 2021, a ban on all direct commercial and private passenger flights from India and Pakistan was implemented throughout Canada (*21*,*22*). Most (82.4%; n = 126) international travel–related introductions of the Delta variant occurred before May 3, 2021 (accounting for day 10 postarrival qPCR testing for persons arriving in Canada before April 23) (Figure 2). At least half (50.8%; n = 64) of international travel–related Delta cases with specimen collection date before May 3, 2021, were in persons arriving from India, whereas 2 (7.4%) of 27 international travel–related Delta cases with specimens collected on or after May 3, 2021, were in persons who were known to have traveled from India (Table 2).

**Table 2:** Information on country of origin for 153 SARS-CoV-2 Delta variant of concern cases reported in British Columbia, Canada, with known history of international travel in periods before and after travel ban and overall, March 1–June 30, 2021

Country of origin	No. (%) Delta cases with international travel history		
	Pre–travel ban period*	Post–travel ban󠆣 period†	Overall
India	64 (50.8)	2 (7.4)	66 (43.1)
Other country	11 (8.7)	15 (55.6)	26 (17.0)
Missing information	51 (40.5)	10 (37.0)	61 (39.9)
Total for all Delta cases	126 (45.2)	27 (3.0)	153 (13.0)

*The Canadian travel ban on direct flights from India and Pakistan was implemented on the evening of April 22, 2021. Persons arriving in Canada before April 23, 2021, completed quantitative PCR testing 10 d after arrival. Therefore, pre–travel ban cases include Delta variant cases with specimen collection during March 21–May 2, 2021. †Delta variant cases with specimen collection during May 3–June 30, 2021.

### Counterfactual Modeling

**Figure 3:**
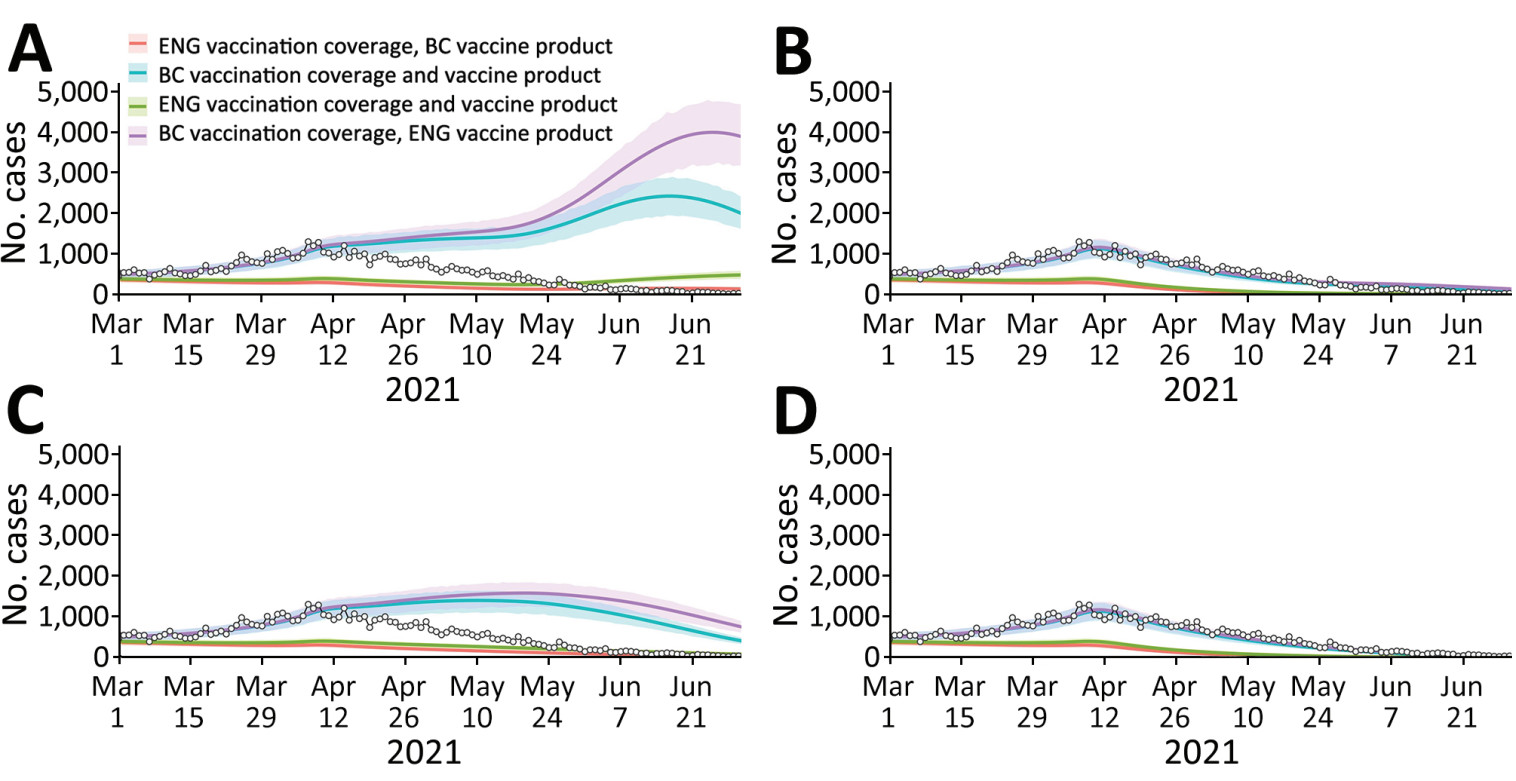
Retrospective counterfactual modeling of COVID-19 transmission in BC, Canada, March 1–June 30, 2021, compared with information from England. Model fitting for March 1, 2020, to July 12, 2021. A) England public health measures and proportion of Delta; B) BC public health measures and England proportion of Delta; C) England public health measures and BC proportion of Delta; D) BC public health measures and proportion of Delta. Each panel represents transmission scenarios derived from 1,000 variational Bayes samples, where measures that affect transmission and the proportion of Delta are reflective of BC or England. Each median line and 90% projection interval shading within each panel represents the vaccination scenario (i.e., population vaccine coverage and majority vaccine product of BC or England). The reported COVID-19 cases for BC are overlaid on each figure as white circles. BC, British Columbia.

Vaccine scheduling and coverage (i.e., timing of vaccination campaign rollout and percentage of population vaccinated) equivalent to that in England resulted in a lower counterfactual COVID-19 case rate in British Columbia than was observed across the study period, irrespective of vaccine product, NPIs, or proportional growth of the Delta variant (Figure 3; Appendix Figure 2). Modeled COVID-19 cases lowered further under the counterfactual scenario in which England’s vaccine scheduling/coverage was combined with the British Columbia majority vaccine product. Within all NPI and proportion-of-Delta scenarios examined, modeled cases were lowest under England’s vaccination scheduling/coverage combined with British Columbia’s majority vaccination product (BNT162b2/mRNA-1273) and highest under British Columbia’s vaccination coverage with England’s majority vaccination product (ChAdOx1) (Figure 3).

Modeling indicates that, without the additional NPIs implemented at the end of March 2021 in British Columbia (Figure 3, panels A, C), a substantially higher COVID-19 caseload would have occurred in British Columbia under the province’s vaccination schedule and coverage, especially if the proportional increase in Delta cases that occurred in England had occurred in British Columbia (Figure 3, panel A). Under England’s vaccine scheduling and coverage, modeled British Columbia cases were still higher than those reported from June 2021 onward without British Columbia’s additional NPIs if England’s proportional increase of Delta had occurred (Figure 3, panel A). In the counterfactual scenario in which England’s proportional increase of Delta occurred in the context of British Columbia’s NPIs and vaccine coverage (Figure 3, panel B), modeled COVID-19 case rates were only slightly higher than reported and much lower than without British Columbia’s NPIs (i.e., compared with Figure 3, panel A). Modeled COVID-19 case rates were lowest under British Columbia’s NPI scenario and proportional increase of Delta across all vaccination scenarios (Figure 3, panel D).

## Discussion

The Delta VOC was first detected in British Columbia during March 21–27, 2021 (epiweek 12); the earliest cases were linked to international travel. Although the Delta variant had already been seeded in the community by the time the countrywide travel ban on direct flights to Canada from India was put in place (*21*), the ban appears to have reduced the number of travelers arriving from countries affected early by the Delta variant, thereby decreasing additional introductions (*23*). This targeted approach might have helped to slow early Delta variant growth in British Columbia, allowing time to increase population vaccination coverage (*23*).

Most Delta variant case-patients in our study were unvaccinated; 14% were partially vaccinated and 3% were fully vaccinated. Those proportions are reflective of the study period, during which the vaccination campaign in British Columbia was primarily focused on first dose rollout: population dose 1 coverage in British Columbia increased from 5% to 77% during the study period, but dose 2 coverage had only reached 28% by the end of the study. In British Columbia, vaccination rollout was primarily prioritized by age (*14*) and most Delta case-patients were young (<40 years). Studies have shown reduced vaccine effectiveness against symptomatic infection or high viral burden for Delta compared with Alpha (*24*,*25*), reiterating the importance of maximizing multidose coverage to improve conferred protection. On the basis of our counterfactual model, earlier population vaccine rollout akin to that done in England, which resulted in higher population dose 1 coverage (39%) by the start of the study period and 57% dose 2 coverage by the end (*15*), would likely have further decreased COVID-19 cases in British Columbia over the study period.

Our counterfactual modeling results suggest that the restrained early growth of COVID-19 cases in British Columbia after Delta was introduced was mainly because of decreased rates of contact from additional NPIs implemented 9 days after the first Delta case was detected, rather than from higher dose 1 vaccine coverage among younger persons or use of mRNA-based vaccines in British Columbia. Our findings are in line with those of McCrone et al. (*26*), who found that the key predictor for higher Delta growth rates between regions in England was increased levels of contact from population mobility and mixing because of the relaxation of NPIs. Results from a survey on behavioral and contact patterns in British Columbia (*27*) indicate that, whereas rates of contact during March–May 2021 either decreased or remained steady for all ages, contact rates increased in all age groups other than persons >65 years of age beginning in June 2021, coinciding with British Columbia’s phased reopening (Appendix Figure 3).

Numerous studies have found NPIs to be associated with reduced COVID-19 transmission and thereby reduced illness, deaths, and strain on healthcare systems (*23*,*28*). Indeed, after a new phase of reopening began in British Columbia on July 1, 2021, including lifting the mask mandate for public indoor spaces and permitting countrywide recreational travel (Appendix Table 6) (*29*), British Columbia experienced a sharp rise in COVID-19 cases (Appendix Figure 4). In the weeks after July 1, 2021 (epiweek 26), a fourth wave of COVID-19 cases occurred in British Columbia, even though population vaccination coverage continued to increase. That wave was driven by the Delta variant, which rapidly grew to dominance, increasing to >70% of weekly incident COVID-19 cases by epiweek 29 (3 weeks later) and >85% by epiweek 30.

The Delta variant was first introduced at a time when British Columbia was experiencing a rise in Alpha and Gamma VOC cases, which required additional NPIs. Dominance of the Delta variant over previous VOCs has been widely reported (*3*,*7*,*8*), but the codominating Alpha and Gamma VOCs in circulation at the time of Delta introduction might have also helped to slow Delta’s initial growth in British Columbia (*30*,*31*), which warrants further exploration. A limitation of this study is that the transmission model used is not multistrain but rather incorporates the increased transmissibility of a variant through modifying the time-varying per-contact transmissibility term to account for increasing prevalence of a more transmissible variant. As such, the time to dominance of a variant is fixed a priori and is not changed by model dynamics; the effect of precirculating strains cannot be fully assessed. The counterfactual model was instead intended to elucidate the effects of the change in the proportion of Delta on the number of reported COVID-19 cases.

Our counterfactual model used a simple modification of the rate of transmission to compare NPIs between British Columbia and England; other differences between the 2 jurisdictions that might have an effect on intercountry comparisons (e.g., demographics and contact patterns) were not considered. Rate of contact and probability of transmission per contact are highly dependent on population density and demographics, social factors, and geographic variation, which were not explicitly captured within these scenarios but were instead fitted to British Columbia reported case data for the 2 transmission scenarios. Hence, the counterfactual model used does not allow for direct comparison of NPI strategies between jurisdictions. Comparison of COVID-19 cases and VOC growth between jurisdictions is further affected by differences in PCR testing rates, as well as VOC detection methods and approach. In this study, we assumed that testing rates were consistent over the study period in each jurisdiction; testing rates in British Columbia (*14*) and England (*32*) did not vary dramatically over this time, indicating relatively consistent case-finding.

In conclusion, spread from returning travelers resulted in community transmission of the emergent Delta VOC in British Columbia beginning in mid-to-late March 2021. However, growth of COVID-19 cases in the initial 3 months after Delta was detected was likely restrained because additional NPIs were implemented soon after variant introduction, including restricting interregional travel and expanding mandatory masking in schools to younger age groups. Our findings highlight the capacity of NPIs to reduce the spread of COVID-19, including highly transmissible variants such as Delta. Maximizing population-level COVID-19 vaccine coverage reduces rates of illness and death and is essential for return to prepandemic ways of life. However, NPIs remain vital for preventing COVID-19–associated burden, especially in the face of variants capable of vaccine escape. Future work should examine the effectiveness of different NPI strategies and the timing of implementing or relaxing NPIs in the context of vaccine coverage, variant-specific vaccine effectiveness, and public acceptance. Identifying the best balance of NPIs to achieve least restrictive means will minimize unintended social, economic, and health-related harms.

Appendix Additional information on emergence of SARS-CoV-2 Delta variant and effect of nonpharmaceutical interventions, British Columbia, Canada.
