# Supplementary material for: Emergence of SARS-CoV-2 Delta Variant and Effect of Nonpharmaceutical Interventions, British Columbia, Canada
Source: Emerg Infect Dis. 2023 Oct;29(10):1999–2007. doi: 10.3201/eid2910.230055 (PMC10521616; doi:10.3201/eid2910.230055)
Supplement: Appendix — Additional information on emergence of SARS-CoV-2 Delta variant and effect of nonpharmaceutical interventions, British Columbia, Canada. [file 23-0055-Techapp-s1.pdf]

Article DOI: https://doi.org/10.3201/eid2910.230055

EID cannot ensure accessibility for supplementary materials supplied by authors. Readers who have difficulty accessing supplementary content should contact the authors for assistance.

Emergence of SARS-CoV-2 Delta Variant and Effect of Nonpharmaceutical Interventions, British Columbia, Canada

Appendix

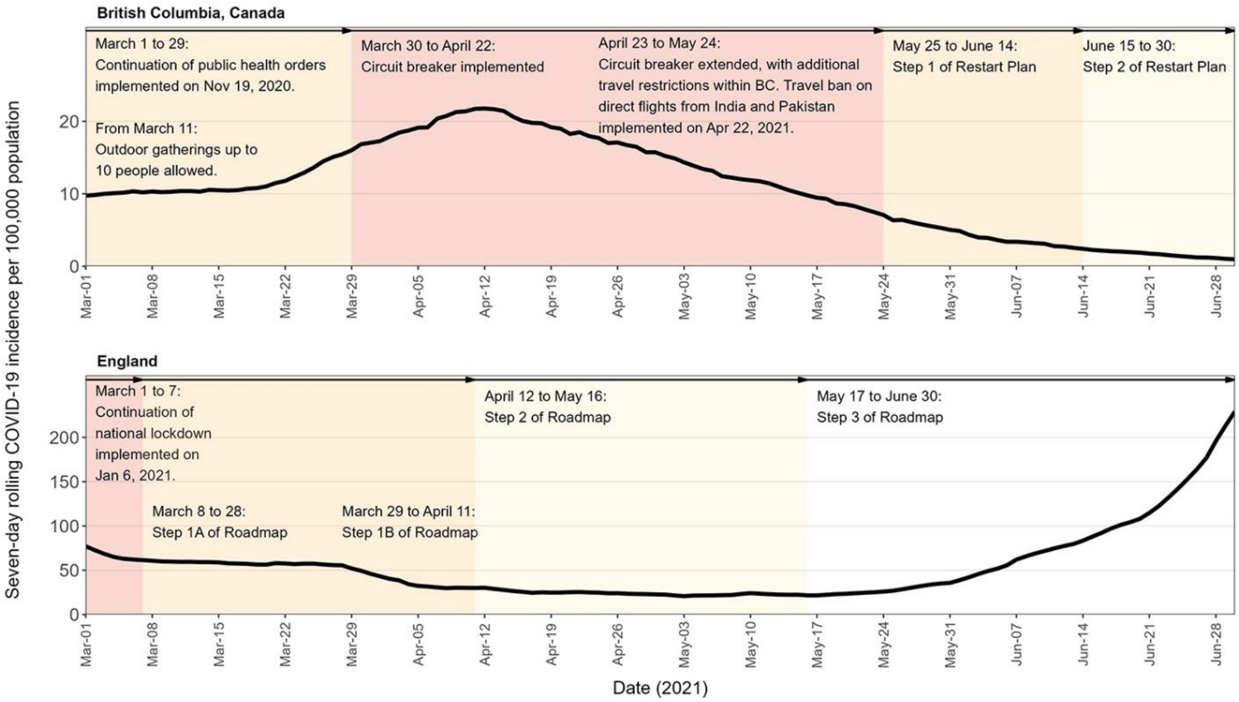

Appendix Figure 1. Seven-day rolling COVID-19 incidence rate per 100,000 people and select non-pharmaceutical interventions in place in British Columbia, Canada and England, March 1 to June 30, 2021. See Appendix Table 1 for more information on changes in public health and social measures, or non-pharmaceutical interventions, implemented per jurisdiction over the study period. Data for 7-day rolling COVID-19 incidence rate for England extracted from UK Health Security Agency COVID-19 dashboard (*1*). BC, British Columbia; VOC, variant of concern.

Appendix Table 1. Timeline of nonpharmaceutical interventions in place in British Columbia, Canada and England, March 1 to June 30, 2021

British Columbia (BC)	England
**Time period: March 1 to March 29, 2021**	**Time period: March 1 to March 7, 2021**
Continuation of mandatory public health orders first implemented in BC on November 19, 2020 (*2–4*):	Continuation of England's third national lockdown first implemented on January 6, 2021 (*5*):
• Physical distancing and masks required in public indoor settings	• Stay-at-home order other than for essential reasons, including essential medical needs, food shopping, exercise, and work if cannot be done from home
• All school staff and middle and secondary school students required to wear non-medical masks indoors (other than when at their desks or workstations)	• Outdoor exercise can be done with your household or bubble, or with 1 person from another household
Personal gatherings:	• All schools, colleges, and universities generally closed or switched to remote learning, with some exceptions (e.g., university students taking hands-on subjects such as medicine)
• No indoor gatherings with people outside immediate household or core bubble (maximum of two people outside household)	• Early years settings (e.g., nurseries) remain open
• Starting March 11: outdoor gatherings with people outside household or core bubble of up to 10 people allowed (*6*)	• Indoor worship services continue, with social distancing
Organized gatherings:	• Recreational facilities and outdoor sports venues closed
• All indoor and outdoor events are prohibited, with the exception of baptisms, funerals, and weddings with up to 10 people including an officiant*; no associated receptions in any venue	• Amateur team sports not allowed
• Indoor worship services suspended; limited outdoor worship services allowed	• Restaurants only offering takeaway meals or food delivery; no indoor dining
Travel:	**Time period: March 8 to March 28, 2021**
• All non-essential travel outside of one's community strongly discouraged	Step 1A of UK's Roadmap out of lockdown begins (*7*):
Businesses, offices, and workplaces:	• Primary and secondary schools and colleges reopen with outdoor after-school sports and activities allowed
• Indoor and outdoor dining allowed with own household or core bubble, up to 6 people per table	o Secondary school and college students and staff required to wear face masks indoors, with voluntary twice-weekly at-home COVID-19 testing
• All businesses and workplaces must conduct active daily screening of all workers using their COVID-19 Safety Plans, and masks must be worn in common areas	o Primary school staff expected to wear face masks in corridors and communal areas where social distancing between adults not possible
• Offices should support work-from-home options wherever possible	• University students on practical courses may also return to in-person learning
Sports and exercise:	**Time period: March 29 to April 11, 2021**
• Indoor group low intensity exercise (e.g., Pilates, hatha yoga) allowed	Step 1B of Roadmap (*8*):
• Indoor group high intensity exercise (e.g., dance, spin, power yoga, circuit training) not permitted	*Personal gatherings:*
• Indoor and outdoor team sports not permitted	• Outdoor gatherings of up to 6 people or 2 households allowed
**Time period: March 30 to April 22, 2021**	*Travel:*
Circuit breaker starts (initially March 30-April 19) (*9*):	• Stay-at-home order replaced with recommendation to "stay local"
• All school staff and students down to Grade 4 now required to wear masks while indoors at school (including at their desks) and on school buses; masks strongly encouraged for students in kindergarten to grade 3	*Sports and exercise:*
Personal gatherings:	• Outdoor sports facilities reopen
• Indoor gatherings continue to be restricted	• Amateur organized team sports allowed, and not subject to the limits on gatherings
• Outdoor gatherings of up to 10 people continue to be allowed	**Time period: April 12 to May 16, 2021**
Organized gatherings:	Step 2 of Roadmap (*10*):
• Indoor worship services suspended. Outdoor worship services may continue.	*Businesses:*
Travel:	• Non-essential retail and personal care open
• Travel within province limited to essential travel only (e.g., work or medical purposes)	• Public buildings (e.g., libraries, community centers) open
• Whistler-Blackcomb ski resort closed, to address and prevent community spread related to non-essential travel	• Self-contained holiday accommodation open
Businesses:	• Outdoor hospitality open
• Indoor dining suspended	• Outdoor attractions and settings open
• Patios remain open, for dining with immediate household or core bubble only	*Sports and exercise:*

British Columbia (BC)	England
Offices and workplaces:	• Indoor leisure facilities (e.g., gyms, swimming pools) open for individuals or within-household groups
• All workers strongly encouraged to work from home, where possible	• All indoor children's activities, including sport, resume
Sports and exercise:	• Indoor parent and child groups of up to 15 people (not including children <5 y) resume
• Indoor, adult group fitness activities suspended	**Time period: May 17 to June 30, 2021**
• Gyms and fitness centers restricted to individual or one-on-one activities	Step 3 of Roadmap (*11*):
**Time period: April 23 to May 24, 2021**	• Face coverings no longer required in schools
Circuit breaker extended on April 19, with additional travel restrictions implemented April 23 (*12*):	*Personal gatherings:*
• Continuation of circuit breaker measures put in place on March 30, 2021	• Indoor gatherings allowed for up to 6 people or 2 households
• Additional travel restriction enforcement within province to further discourage non-essential travel	• Outdoor gatherings allowed for up to 30 people
The Government of Canada implemented a travel ban on direct flights from India and Pakistan beginning the evening of April 22, 2021.	*Organized gatherings:*
**Time period: May 25 to June 14, 2021**	• Outdoor sporting events allowed up to 4,000 people or at 50% of venue's capacity, whichever is lower. Large outdoor seated venues, where crowds can be distributed, allowed up to 10,000 people or 25% of total seated capacity, whichever is lower
Step 1 of BC's Restart Plan (*13*):	• Increased attendance allowed at events such as weddings and funerals (e.g., weddings allowed to have more than 30 guests from June 21st)
• Physical distancing and masks continue to be required in public indoor settings	*Travel:*
Personal gatherings:	• International travel to "green list" countries resumes
• Indoor gatherings of up to 5 people or 1 other household	*Businesses:*
• Outdoor gatherings of up to 10 people continue	• Indoor hospitality, entertainment venues, and hotels open
Organized gatherings:	*Offices and workplaces:*
• Indoor seated gatherings of up to 10 people*	• Continued recommendation to work from home if possible
• Outdoor seated gatherings (e.g., weddings) of up to 50 people*	*Sports and exercise:*
• Indoor or outdoor worship services of up to 50 people*	• Adult indoor group sports and exercise classes resume
Travel:	Step 4 postponed from June 21st until after the study period due to rising Delta variant cases.
• Recreational travel within own travel zone	
• Non-essential travel between the government's three designated travel zones still restricted	
Businesses:	
• Indoor and outdoor dining for groups up to 6 people, not restricted to own household or core bubble	
• Liquor served until 10pm	
Offices and workplaces:	
• Start of gradual return to workplaces	
• Employers must continue to have a COVID-19 Safety Plan and daily health check in place	
Sports and exercise:	
• Low-intensity indoor fitness classes (e.g., Pilates) resume with limited capacity	
• Outdoor team sports are allowed for all ages, without spectators	
**Time period: June 15 to June 30, 2021**	
Step 2 of BC's Restart Plan (*14*):	
• Physical distancing and masks continue to be required in public indoor settings.	
Personal gatherings:	
• Outdoor gatherings of up to 50 people (e.g., birthday parties, backyard BBQs, block parties)	
• Indoor gatherings of up to 5 people or 1 other household	
Organized gatherings:	
• Indoor seated gatherings of up to 50 people*	
• Outdoor gatherings of up to 50 people*	
• Indoor or outdoor worship services of up to 50 people*	
Travel:	
• Recreational travel within BC	
Businesses:	

British Columbia (BC)	England
• Indoor and outdoor dining for groups up to 6 people, not restricted to own household or core bubble<br>• Liquor served until midnight<br>• Banquet halls reopen with limited capacity*<br>*Offices and workplaces:*<br>• Continued return to workplaces<br>• Small, in-person meetings allowed<br>• Employers must continue to have a COVID-19 Safety Plan and daily health check in place<br>*Sports and exercise:*<br>• Indoor high- and low-intensity group exercise allowed with reduced capacity<br>• Indoor and outdoor team sports allowed for all ages<br>• Up to 50 spectators allowed outdoors, no spectators at any indoor sport activities	

*With an approved BC COVID-19 Safety Plan.

Appendix Table 2. Combined screening and detection strategy for variants of concern in British Columbia, Canada, March 1 to June 30, 2021*

Dates	VOC detection strategy
**March 1 to May 29, 2021**	Combined VOC testing strategy using targeted VOC SNP qPCR ("screening") and whole-genome sequencing (WGS):
	• Under the combined testing strategy, ≈80 to 99% of positive samples were screened for VOCs each week; a subset of screened cases were further confirmed by WGS. See below for details on screening methods and proportion of screened cases further confirmed by WGS, by time period.
	• A separate subset of positive samples underwent WGS, including: a random sample for baseline surveillance, those associated with clusters/outbreaks, hospitalization, re-infection, and vaccine breakthrough, and those associated with international travelers arriving at the BC border.
**March 1 to 31, 2021:**	VOC SNP qPCR screening methods in BC primarily only detected the N501Y mutation (present in Alpha, Beta, and Gamma VOCs). One hospital in BC implemented K417T mutation screening for Gamma VOC detection from March 21, 2021 onwards. During this time period, all presumptive positive SNP qPCR results were confirmed by WGS at the BCCDC PHL.
**April 1 to May 29, 2021:**	VOC SNP qPCR screening methods were modified to incorporate both N501Y and E484K mutation screening at the BCCDC PHL (E484K mutation is detected in Beta and Gamma VOCs, but rarely in Alpha VOC). Specimens that tested positive for N501Y alone were identified as presumptive B.1.1.7 lineage; ~10% were confirmed by WGS. In addition, only ~25% of specimens that tested positive for N501Y and another mutation were confirmed by WGS. Based on epidemiology at the time, VOC screening results with both E484K and N501Y mutations were assumed to be Gamma VOC, given the very low prevalence of Beta VOC in BC.
**May 30 to June 30, 2021**	All positive samples in BC had WGS attempted.

*BC, British Columbia; BCCDC PHL, British Columbia Centre for Disease Control Public Health Laboratory; SNP qPCR, single-nucleotide polymorphism quantitative PCR; VOC, variant of concern; WGS, whole-genome sequencing.

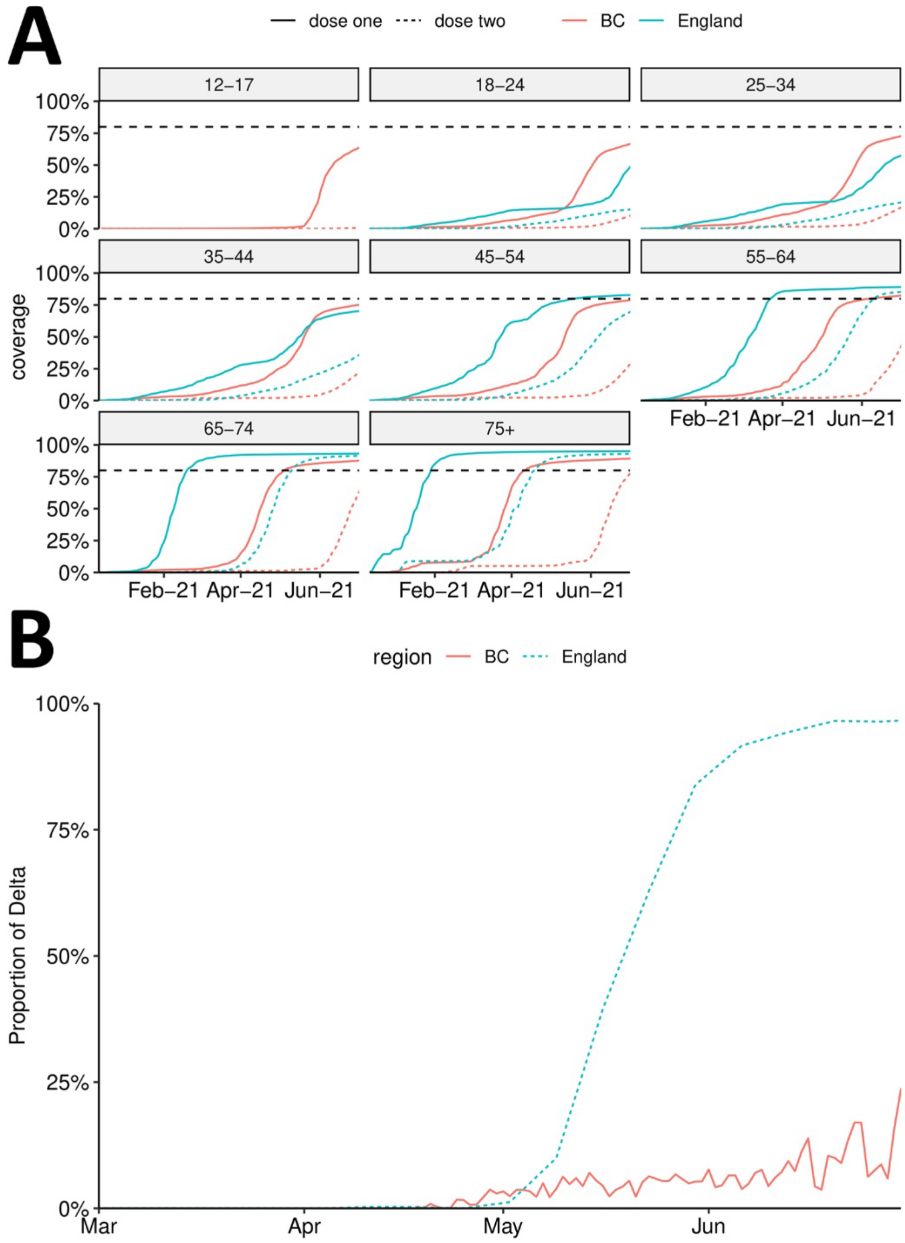

Appendix Figure 2. Comparison of England and British Columbia vaccination coverage (A) and proportion of genotyped cases identified as Delta variant of concern (B). Vaccine dose coverage includes the coverage of both dose one and dose two by age group, with the 80% coverage highlighted as a dashed line for clarity. Population vaccination coverage data were extracted from the UK Government COVID-19 dashboard (*15*) for England and from the Provincial Immunization Registry for BC. Note that England had not started vaccination of the adolescent group (ages 12–17) before July 2021. Data for the Delta variant in England was extracted from the UK Health Security Agency Technical Briefing 19 (*16*) of all available genotyped cases. British Columbia Delta variant prevalence was extracted from all cases genotyped through screening and background surveillance.

Appendix Table 3. Case definitions for variants of concern*

VOC	Case definition (* denotes any number indicating a sub-lineage)†
**Delta**	SARS-CoV-2-positive cases whose samples were confirmed through whole-genome sequencing as Pangolin lineage B.1.617.2 or AY.*
**Alpha**	SARS-CoV-2-positive cases whose samples were confirmed through whole-genome sequencing as Pangolin lineage B.1.1.7 or Q.*, or screened as N501Y-positive and E484K-negative
**Gamma**	SARS-CoV-2-positive cases whose samples were confirmed through whole-genome sequencing as Pangolin lineage P.1 or P.1.*, screened as N501Y- and E484K-positive, or screened as K417T-positive
**Beta‡**	SARS-CoV-2-positive cases whose samples were confirmed through whole-genome sequencing as Pangolin lineage B.1.351 or B.1.351.*

*VOC, variant of concern.
†Screening employed targeted single-nucleotide polymorphism quantitative polymerase chain reaction (SNP qPCR).
‡Based on epidemiology at the time, VOC screening results with both E484K and N501Y mutations were assumed to be Gamma VOC, given the very low prevalence of Beta VOC in BC.

Appendix Table 4. Modeling approach and parameters used for each counterfactual scenario

Scenario	Modeling approach	Parameters and explanation
Baseline	Baseline transmission was fit using a Bayesian approach to daily BC PCR-confirmed COVID-19 cases. Transmission was modeled as a piecewise constant function with pre-specified breakpoints.	Breakpoints were selected based on changes in public health measures or population behavior. The start dates for each segment were: 2020–03–15, 2020–05–10, 2020–09–08, 2020–09–21, 2020–11–10, 2021–01–25, 2021–03–29, 2021–04–05, 2021–05–25.
Introduction of Delta variant	Logistic growth curve multiplied by transmission rate β to replicate the relative increase in transmission as the Delta variant became dominant. The parameters include the time of introduction and the time to the proportion of Delta being 90% among all variants.	The BC scenario ramp had a start date of 2021–05–25 and a time to dominance of 25 weeks. The England scenario had a start date of 2021–05–01 and a time to dominance of 4 weeks to reflect the differences in the proportional change in Delta between both jurisdictions. For each scenario, Delta increased transmission by 50% (*17*).
Public health measures and health behavior	A further change in the transmission was included to characterize the changes in public health measures and behavior following their introduction on 2021–04–05 in BC. This was modeled as a step-change in the transmission term after implementation of the measures.	In the BC scenario, the transmission term was fitted to historical data and so no further changes needed to be included. For the England scenario, the transmission term is multiplied by a factor of 50% reflecting no changes in public health measures following 2021–04–05.
Vaccination roll-out and coverage	The exact proportion of vaccination by age group were extracted for both BC and England jurisdictions. For each vaccination coverage, an estimate of BC's population structure and contact rates by age group were used to derive an age-adjusted vaccination rate for each scenario.	Proportion of each age group vaccinated based on data for BC and England. Number of weekly contacts per age group based on BC-Mix survey data (*18*) were: <2 y: 6, 2–5 y: 12, 6–17 y: 13, 18–24 y: 44, 25–34 y: 44, 35–44 y: 48, 45–54 y: 48, 55–64 y: 24, 65–74 y: 21, >75 y: 17.
Vaccination product	The vaccine transmission-blocking effectiveness was selected for each scenario based on the dominant vaccine product for each jurisdiction during the study period. In BC this was BNT162b2 and in England this was ChAdOX1.	Vaccine effectiveness for the BC scenario was 71.8% after one dose and 89% after two doses. Vaccine effectiveness for the England scenario was 58% after one dose and 77% after two doses.

*BC, British Columbia.

Appendix Table 5. Transmission-blocking efficacy parameters used in counterfactual modeling by vaccine product and first and second dose

Vaccine product	Dose one	Dose two
BNT162b2 (Pfizer)	71.8%	89%
ChAdOx1 (AstraZeneca)	58%	77%

*Efficacy parameters for vaccination incorporated both a reduction in the probability of incidence infection as well as reduction in onward transmission if infected while vaccinated. Estimates for vaccine transmission-blocking efficacy and onward-transmission efficacy were derived from values presented in the UK Scientific Pandemic Influenza Group on Modeling, Operational subgroup (SPI-M-O) of the Scientific Advisory Group for Emergencies (SAGE) July 7th modeling group report (*19*) where a range of optimistic to more pessimistic vaccine assumptions were provided by different modeling groups. The total transmission blocking probability is 1-(1–0.6)*(1–0.45) = 0.78 under the optimistic scenario and 1-(1–0.53)*(1–0.4) = 0.718 under the pessimistic scenario for BNT162b2, rising to 0.89 under the second dose. For ChAdOx1, after one dose, this is 0.58 under the pessimistic and 0.65 under the optimistic scenarios, rising to 0.77 under the second dose pessimistic and 0.8 under the optimistic scenarios, respectively. Scenarios in British Columbia were more in line with pessimistic assumptions for BNT162b2, so for ChAdOx1 we also selected the pessimistic assumption.

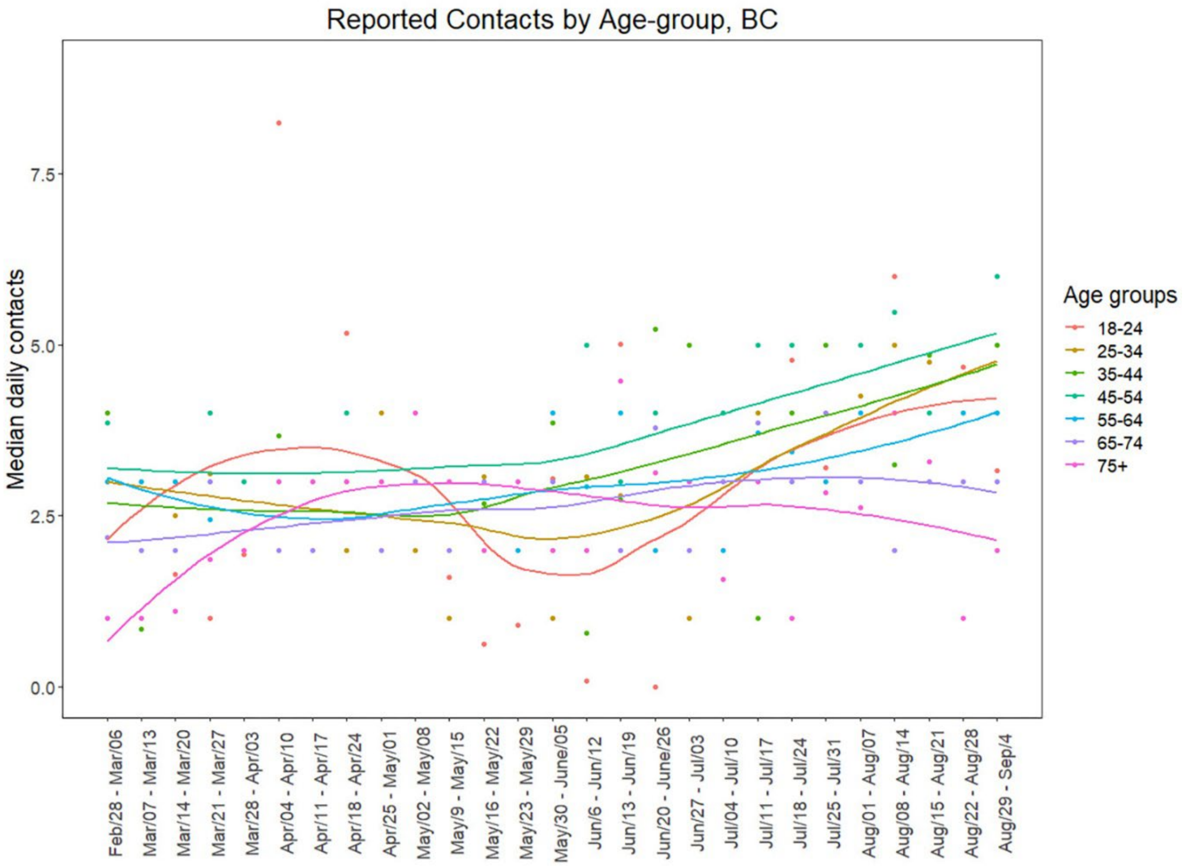

Appendix Figure 3. Sample median number of contacts by age group in British Columbia, Canada, February 28 to September 4, 2021. Reported contacts by age group in BC extracted from BC Mix COVID-19 Survey data (*18*). Fitted lines constructed using local polynomial regression with LOESS method.
BC, British Columbia.

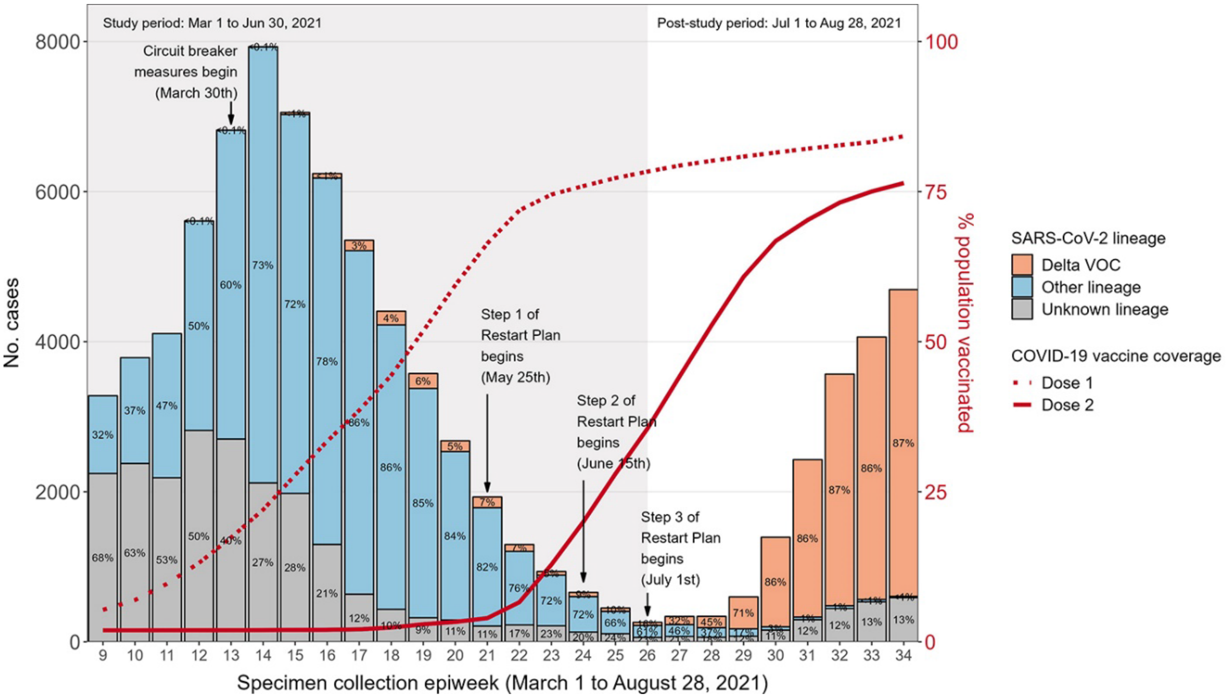

Appendix Figure 4. COVID-19 cases in British Columbia, Canada by Delta variant of concern, other lineage, or unknown lineage, with specimen collection date between March 1 and August 28, 2021, overlaid with 12 year and older population COVID-19 vaccination coverage by dose received. Step 3 of BC's Restart Plan included lifting of the mask mandate in indoor public spaces, removal of limits on indoor and outdoor personal gatherings, increased capacity for indoor and outdoor organized gatherings, allowing of Canada-wide recreational travel, increased capacity at indoor sports and exercise facilities, re-opening of casinos and nightclubs, and return to normal hours for liquor service at restaurants, bars and pubs, with no group limits for tables (*20*) (Appendix Table 6). BC, British Columbia; VOC, variant of concern.

Appendix Table 6. Changes to public health and social measures in British Columbia, Canada following July 1, 2021 move to Step 3 of provincial Restart Plan

British Columbia
Implemented on July 1, 2021
Step 3 of BC's Restart Plan (*20*):
• Masks recommended until fully vaccinated, but not mandatory, in public indoor spaces
• Physical distancing remains in place
Personal gatherings:
• No limits on personal gathering sizes indoors or outdoors
Organized gatherings:
• Indoor organized gatherings of up to 50 people, or 50% capacity, allowed with a COVID-19 Safety Plan
• Outdoor organized gatherings of up to 5,000 people, or 50% capacity, allowed with a COVID-19 Safety Plan
• Fairs, festivals, and trade shows return to normal, with Communicable Disease Plan in place
Travel:
• Recreational travel within Canada allowed
Businesses, offices, and workplaces:
• No limits on group sizes for indoor or outdoor dining
• Return to regular liquor service
• Bingo halls, casinos, and nightclubs reopen with reduced capacity
• Seminars and bigger meetings allowed for offices and workplaces
Sports and exercise:
• Return to normal for sports and exercise facilities, with Communicable Disease Plan in place
While BC's state of public health emergency remained in effect to support amended public health orders from the provincial health officer, Step 3 of BC's Restart Plan also signaled the end of BC's provincial state of emergency in response to the COVID-19 pandemic, which had been in place since March 2020 (*20*). The provincial state of emergency had granted the BC government additional powers such as the ability to enforce the mandatory mask policy and travel restrictions that had been in place.

*BC, British Columbia.

References

1. UK Health Security Agency. Coronavirus (COVID-19) in the UK: Cases in England [cited 2022 Feb 1]. https://coronavirus.data.gov.uk/details/cases?areaType=nation&areaName=England

2. BC Ministry of Health. Joint statement on B.C.'s COVID-19 response, latest updates [cited 2022 Dec 10]. https://news.gov.bc.ca/releases/2020HLTH0061-001949

3. British Columbia Ministry of Health. Public Health Guidelines for Group Low Intensity Exercise [cited 2022 Dec 10]. https://www2.gov.bc.ca/assets/gov/health/about-bc-s-health-care-system/office-of-the-provincial-health-officer/covid-19/covid-19-public-health-guidelines-low-intensity-exercise.pdf?bcgovtm=20200506_GCPE_AM_COVID_9_NOTIFICATION_BCGOV_BCGOV_EN_BC__NOTIFICATION

4. Ministry of Education. Enhanced safety measures for K-12 schools [cited 2022 Dec 10]. https://news.gov.bc.ca/releases/2021EDUC0011-000201

5. Prime Minister's Office. 10 Downing Street and The Rt Hon Boris Johnson MP. Press release: Prime Minister announces national lockdown [cited 2022 Dec 10]. https://www.gov.uk/government/news/prime-minister-announces-national-lockdown

6. BC Ministry of Health. Joint statement on B.C.'s COVID-19 response, latest updates [cited 2022 Dec 10]. https://news.gov.bc.ca/releases/2021HLTH0020-000457

7. Cabinet Office. Guidance: COVID-19 Response – Spring 2021 (Summary) [cited 2022 Dec 10]. https://www.gov.uk/government/publications/covid-19-response-spring-2021/covid-19-response-spring-2021-summary

8. Prime Minister's Office. 10 Downing Street and Department of Health and Social Care. COVID-19: Restrictions in England From 29 March [cited 2022 Dec 10]. https://www.gov.uk/government/publications/covid-19-restrictions-posters-29-march-2021

9. Office of the Premier. Three-week circuit breaker begins now to bend the curve, protect people [cited 2022 Dec 10]. https://news.gov.bc.ca/releases/2021PREM0023-000578

10. Prime Minister's Office. 10 Downing Street. Press release: Further easing of Covid restrictions confirmed for 12 April [cited 2022 Dec 10]. https://www.gov.uk/government/news/further-easing-of-covid-restrictions-confirmed-for-12-april

11. Prime Minister's Office. 10 Downing Street. Press release: Further easing of COVID restrictions confirmed for 17 May [cited 2022 Dec 10]. https://www.gov.uk/government/news/further-easing-of-covid-restrictions-confirmed-for-17-may

12. BC Ministry of Health. Joint statement on B.C.'s COVID-19 response, latest updates [cited 2022 Dec 10]. https://news.gov.bc.ca/releases/2021HLTH0079-000742

13. Office of the Premier. B.C. launches restart plan to safely bring people back together [cited 2022 Dec 10]. https://news.gov.bc.ca/releases/2021PREM0037-001008

14. Office of the Premier. B.C. prepares to safely move to Step 2 of its restart plan [cited 2022 Dec 10]. https://news.gov.bc.ca/releases/2021PREM0041-001155

15. UK Health Security Agency. Coronavirus (COVID-19) in the UK: Vaccinations in England [cited 2021 Aug 6]. https://coronavirus.data.gov.uk/details/vaccinations?areaType=nation&areaName=England

16. Public Health England. SARS-CoV-2 variants of concern and variants under investigation in England: Technical briefing 19 [cited 2021 Aug 6]. https://assets.publishing.service.gov.uk/government/uploads/system/uploads/attachment_data/file/1005517/Technical_Briefing_19.pdf

17. Scientific Advisory Group for Emergencies. SPI-M-O: Consensus statement on COVID-19, 3 June 2021 [cited 2022 May 26]. https://www.gov.uk/government/publications/spi-m-o-consensus-statement-on-covid-19-3-june-2021

18. Adu P, Binka M, Mahmood B, Jeong D, Buller-Tylor T, Jean Damascene M, et al. Quantifying contact patterns: development and characteristics of the British Columbia COVID-19 Population Mixing Patterns Survey [cited 2022 Dec 10]. Int J Infect Dis. 2022;116:S30–1. https://doi.org/10.1016/j.ijid.2021.12.073

19. Scientific Advisory Group for Emergencies. SPI-M-O: Summary of further modelling of easing restrictions - Roadmap Step 4 on 19th July 2021 [cited 2021 Aug 6]. https://assets.publishing.service.gov.uk/government/uploads/system/uploads/attachment_data/file/1001169/S1301_SPI-M-O_Summary_Roadmap_second_Step_4.2__1_.pdf

20. Office of the Premier. B.C. shifts to Step 3 of restart plan [cited 2022 Dec 10]. https://news.gov.bc.ca/releases/2021PREM0043-001268
